# Molecular characterization of field resistance to Fusarium head blight in two US soft red winter wheat cultivars

**DOI:** 10.1007/s00122-013-2149-y

**Published:** 2013-07-06

**Authors:** Shuyu Liu, Carl A. Griffey, Marla D. Hall, Anne L. McKendry, Jianli Chen, Wynse S. Brooks, Gina Brown-Guedira, David Van Sanford, David G. Schmale

**Affiliations:** 1 Department of Crop and Soil Environmental Sciences, Virginia Tech, Blacksburg, VA 24060 USA; 2 Department of Plant Science, University of Missouri, Columbia, MO 65201 USA; 3 Eastern Regional Small Grains Genotyping Lab, USDA-ARS, Raleigh, NC 27695 USA; 4 Department of Plant and Soil Sciences, University of Kentucky, Lexington, KY 40546 USA; 5 Department of Plant Pathology, Physiology and Weed Science, Virginia Tech, Blacksburg, VA 24060 USA; 6 Present Address: Texas A&M AgriLife Research and Extension Center, Texas A&M University System, 6500 Amarillo Blvd W., Amarillo, TX 79106 USA; 7 Present Address: Limagrain Cereal Seeds, 6414 N. Sheridan, Wichita, KS 67204 USA; 8 Present Address: Department of Agronomy, University of Idaho Aberdeen Research and Extension Center, Aberdeen, ID 83210 USA

## Abstract

In the soft red winter wheat (*Triticum aestivum* L.) regions of the US, Fusarium head blight (FHB, caused by *Fusarium* spp.) resistance derived from locally adapted germplasm has been used predominantly. Two soft red winter wheat cultivars, Massey and Ernie, have moderate resistance to FHB. Mapping populations derived from Becker/Massey (B/M) and Ernie/MO 94-317 (E/MO) were evaluated for FHB resistance and other traits in multiple environments. Eight QTL in B/M and five QTL in E/MO were associated with FHB variables including incidence, severity (SEV), index (IND), Fusarium damaged kernels (FDK), deoxynivalenol (DON), and morphological traits flowering time and plant height. Four QTL were common to both populations. Three of them were located at or near known genes: *Ppd*-*D1* on chromosome 2DS, *Rht*-*B1* on 4BS, and *Rht*-*D1* on 4DS. Alleles for dwarf plant height (*Rht*-*B1b* and *Rht*-*D1b*) and photoperiod insensitivity (*Ppd*-*D1a*) had pleiotropic effects in reducing height and increasing FHB susceptibility. The other QTL detected for FHB variables were on 3BL in both populations, 1AS, 1DS, 2BL, and 4DL in B/M, and 5AL (*B1*) and 6AL in E/MO. The additive effects of FHB variables ranged from 0.4 mg kg^−1^ of DON to 6.2 % for greenhouse (GH) SEV in B/M and ranged from 0.3 mg kg^−1^ of DON to 8.3 % for GH SEV in E/MO. The 4DS QTL had epistasis with *Ppd*-*D1*, *Qdon.umc*-*6AL*, and *Qht.umc*-*4BS*, and additive × additive × environment interactions with the 4BS QTL for SEV, IND, and FDK in E/MO. Marker-assisted selection might be used to enhance FHB resistance through selection of favorable alleles of significant QTL, taking into account genotypes at *Rht*-*B1b*, *Rht*-*D1a* and *Ppd*-*D1a*.

**Electronic supplementary material:**

The online version of this article (doi:10.1007/s00122-013-2149-y) contains supplementary material, which is available to authorized users.

## Introduction

Fusarium head blight (FHB), mainly caused by *Fusarium graminearum* Schwabe [teleomorph: *Gibberella zeae* Schw. (Petch)], is a serious disease that reduces grain yield and quality of wheat (*Triticum aestivum* L.) in warm and humid areas worldwide. Growing resistant cultivars is an economically effective and environmentally sound way of managing FHB (Martin and Johnston 1982). Many QTL have been identified in diverse wheat sources worldwide (Buerstmayr et al. 2009; Liu et al. 2009; Loffler et al. 2009). Most resistant sources characterized to date are spring wheat genotypes from Asia and South America. Among winter wheat sources for which resistance QTL have been mapped, most are of European descent [e.g., Sincron and F201R from Romania (Ittu et al. 2000; Shen et al. 2003), Renan from France (Gervais et al. 2003), and Dream, Ritmo, and GS16-92 from Germany (Schmolke et al. 2005; Klahr et al. 2007; Schmolke et al. 2008)]; however, they are not adapted to most of the winter wheat production areas of the US. Currently, only a few soft red winter (SRW) wheat sources from the US including Ernie, Goldfield, Freedom, IL 94-1653, and VA00W-38 (Liu et al. 2005, 2007; Abate et al. 2008; Gilsinger et al. 2005; Gupta et al. 2001; Bonin and Kolb 2009; Liu et al. 2012b) have been genetically characterized for FHB resistance either in greenhouse or field conditions.

Screening for type II resistance (pathogen spread within the spike) under controlled environmental conditions via point inoculation is most reliable for assessing FHB severity (Bai and Shaner 1994). Therefore, many QTL mapping studies have been conducted as greenhouse experiments (Buerstmayr et al. 2009; Liu et al. 2009). However, assessment of FHB resistance expressed under field conditions is more comprehensive and provides information on the different types and overall level of FHB resistance. Many FHB field experiments have been conducted using grain spawn and/or conidia (asexual spores) spray inoculations in mist-irrigated nurseries (Buerstmayr et al. 2009; Liu et al. 2012b). Assessments made in these studies have included FHB incidence (INC) and severity (SEV) with subsequent analysis of FHB index (IND), Fusarium damaged kernels (FDK), and concentrations of deoxynivalenol (DON).

Plant height, heading date, flowering time, and the presence of awns have been reported to be associated with FHB. Previous studies have reported that tightly linked genes or pleiotropic effects of dwarfing genes rather than plant height per se confer increased FHB susceptibility in European wheat cultivars (Draeger et al. 2007; Srinivasachary et al. 2008). FHB resistance QTL were identified overlapping QTL for plant height and flowering time in wheat cultivars Renan and Recital (Gervais et al. 2003). Mesterhazy (1995) reported that wheat lines having long awns were more susceptible to FHB under natural conditions.

The process of identifying and characterizing new sources of FHB resistance in adapted germplasm and developing reliable markers to facilitate marker-assisted selection (MAS) and pyramiding of diverse resistance QTL is critical to the enhancement of FHB resistance in breeding programs. After more than a decade of FHB research on plant introductions, wheat breeders in the SRW wheat region of the US consider the use of FHB resistance from locally adapted sources to be most promising. SRW wheat cultivars with moderate resistance to FHB include Freedom, Ernie, Truman, Bess, Roane, Tribute, and Jamestown (Gooding et al. 1997; McKendry et al. 1995, 2005, 2007; Griffey et al. 2001, 2005, 2010). The current study used recombinant inbred lines (RILs) derived from two moderately FHB-resistant cultivars, Massey and Ernie, in the SRW wheat regions of the US with the following objectives: (1) map QTL associated with FHB resistance in Massey and Ernie using phenotypic data from multiple environments; (2) elucidate the main and epistatic effects of QTL and interactions between QTL and environments and; (3) determine the association between FHB resistance QTL with genes governing plant height, photoperiod sensitivity and awns, and their impact on breeding for FHB resistance.

## Materials and methods

### Plant materials

Massey (*Rht*-*B1b*) was derived from the cross of Blueboy/Knox62 (Starling et al. 1984). Its semi-dwarf allele was derived from line Norin 10 (Murphy 1967). Becker, released by Ohio State University in 1985 (Lafever 1988), was derived from the cross of Hart/VA 66-54-10 (CItr 15293) and has *Rht*-*D1b* and *Rht8c*, which is in coupling linkage with the 192 bp allele of Xgwm261. Massey is sensitive to photoperiod (*Ppd*-*D1b*) while Becker is insensitive (*Ppd*-*D1a*). The initial cross of Becker/Massey was made in 1991. Lines were advanced in bulk to the F_7:14_ generation by 2007. Only 152 of the initial RILs were used in FHB tests in the greenhouse for severity in 2007 and 2008 and in the field FHB nursery in 2008 and 2009 due to heterogeneity of some lines (Liu et al. 2012a).

The wheat cultivar Ernie (*Rht*-*B1b*) has partial resistance to FHB while MO 94-317 (*Rht*-*D1b*) is susceptible (McKendry et al. 1995; Liu et al. 2005, 2007; Abate et al. 2008). Ernie is photoperiod sensitive (*Ppd*-*D1b*) and has the awn suppression gene *B1*. A set of 231 Ernie/MO 94-317 F_11_ RILs were advanced and evaluated in this study.

### Phenotypic screening and data analyses

Parents and RILs of both populations were screened for FHB in multiple environments (Liu et al. 2012a). Eight environments were inoculated and mist-irrigated in the Virginia scab nurseries (VASN) at Blacksburg in 2007 (B/M) and from 2008 to 2009 (B/M, E/MO), Missouri scab nursery (MOSN) at Columbia in 2008 (B/M, E/MO), Virginia greenhouse (VAGH) in 2007 and 2008 (B/M), and greenhouse at Columbia, MO in 2002 and 2003 for E/MO reported by Liu et al. (2007) and Abate et al. (2008). Experiments were also conducted under natural field conditions at Lexington, Kentucky (KYFLD) in 2008 (B/M) and at Warsaw, Virginia (VAFLD) in 2009 (B/M, E/MO). Single 1.2 m head rows with 0.3 m spacing between rows were planted in randomized complete block designs with two replications for all tests except the VAFLD test having one replication of two head rows. Greenhouse experiments were arranged in randomized complete block designs with parents and RILs planted in D40 Deepots (Hummert International, Earth City, MO, USA) and evaluated according to the procedures described in Liu et al. (2007). The most aggressive *F. graminearum* isolates from VA and MO, respectively, were used for spray inoculations (1 × 10^4^ conidia spores per mL) in the field FHB nurseries at flowering time and for point inoculations (5 × 10^4^ conidia spores per mL) of middle florets in greenhouse experiments. In field experiments, data for FHB variables including INC, SEV, IND, FDK and DON were collected following methods of Liu et al. (2012a, b). The data collected from greenhouse followed the procedure by Liu et al. (2007). The concentration of DON was measured using gas chromatography–mass spectrometry following procedures established by Khatibi et al. (2012) and Tacke and Casper (1996). In this study, assessments used to quantify FHB resistance including INC, SEV, IND, FDK, and DON are collectively referred to as FHB variables. Flowering time (FT, days from Jan 1 to 50 % flowering) or heading date, INC, SEV, and IND were assessed in all six field environments except that SEV and IND were not assessed at KYFLD in 2008. FDK and DON were only estimated at VASN in 2008 and 2009 and at VAFLD in 2009. Plant height was recorded in the VASN and VAFLD tests in 2009 and the greenhouse experiment in 2010 as described by Liu et al. (2012a).

Analyses of variance (ANOVA) were conducted in all replicated experiments using PROC GLM (SAS Institute 2008). Correlation coefficients among FHB variables and other traits were calculated for each environment in VASN in 2008 and 2009 and VAFLD in 2009 using SAS PROC CORR.

### Genotypic data

Sample DNA from each parent and RIL of B/M were extracted according to the protocol recommended at http://www.triticarte.com.au/content/DNA-preparation.html and sent to Triticarte Pty Ltd (Yarralumla, Australia) for whole genome Diversity Arrays Technology (DArT) analysis (Akbari et al. 2006). A total of 740 DArT markers were scored. A set of 199 simple sequence repeat (SSR) markers were screened for polymorphism between the parents and 96 SSR markers were used to screen all RILs.

For E/MO RILs, in addition to 94 SSR loci and 45 amplified fragment length polymorphic markers mapped previously (Liu et al. 2007), 120 new SSR markers were tested for polymorphism and a set of 70 additional SSR were added to construct the new chromosome maps.

Primer sequences from GrainGenes 2.0 (http://wheat.pw.usda.gov/GG2/index.shtml, Accessed 1 June 2010) were obtained either as synthesized directly labeled primers with fluorophores (FAM, NED, PET, or VIC) from Applied Biosystems (Carlsbad, CA, USA) or indirectly labeled with a M_13_ tail. Indirectly labeled primers were ordered from Integrated DNA Technology (Coralville, IA, USA). The PCR reaction solution preparation and amplification cycle were same as described by Christopher et al. (2013). The mixed solution of 2 μL PCR product, 8.5 μL of formamide containing 1/66 volume of GeneScan^TM^ 500 Liz^®^ Size Standard from Applied Biosystems (Carlsbad, CA, USA) was visualized via capillary electrophoresis on an ABI 3130xl Genetic Analyzer from Applied Biosystems (Carlsbad, CA, USA). GeneMarker from SoftGenetics, LLC (State College, PA, USA) was used to analyze polymorphic bands across the whole RIL population.

### Construction of genetic map and detection of QTL

Molecular markers were analyzed using Joinmap 3.0 (Van Ooijen and Voorrips 2001) combined with MapMaker 3.0 (Lander et al. 1987) with logarithm of odds (LOD) at 3.0, genetic distance <40 cM, and the Kosambi mapping function (Kosambi 1944). The generated map file and raw data file including markers and phenotypic data were imported into QTL Cartographer 2.0 (Wang et al. 2010) to generate files for further analyses.

QTL Network 2.0 was used to analyze the main additive (A), epistatic effects (A × A) and interaction effects of QTL and environments (A × E, A × A × E) (Yang et al. 2008) across all the tested environments. Each of eight traits (INC, SEV, IND, FDK, DON, GHSEV, FT, and HT) was assessed in between two and six environments for B/M and from between two and four environments for E/MO. A 1,000 permutation test was used to calculate critical *F*-values for an experiment-wise significance level of 0.05. Tests to detect QTL were conducted at 1-cM intervals with a window size of 10 cM (Yang et al. 2008). A Monte Carlo Markov Chain approach was used to estimate QTL effects. The genetic maps and QTL intervals were drawn using MapChart 2.0 (Voorrips 2002).

Group means with various combinations of alleles were analyzed for FHB variables and traits using Tukey’s studentized range (HSD) of multiple comparisons with different sample sizes by SAS PROC GLM at *P* < 0.05.

## Results

### Analysis of FHB variables and traits

Analyses of variance were conducted on data from tests with two replications for FHB variables and morphological traits in the VASN in 2008 and 2009 (Supplementary Table S1). RILs varied significantly (*P* < 0.001) for all FHB variables and traits (INC, SEV, IND, FDK, DON, FT, and HT) with the exception of DON in B/M. Year effects were not significant for SEV and FDK in B/M or for DON in both populations. The interactions between line and year were all significant at *P* < 0.001 except for DON in B/M and FT in E/MO. The results indicated that environmental effects significantly influenced FHB in addition to the major genetic effects.

Parental means and ranges among RILs for five FHB variables and three agronomic traits were analyzed using data from four environments (MOSN in 2008, VASN in 2008 and 2009, VAFLD in 2009) for B/M (Liu et al. 2012a) and E/MO (Data not shown). Transgressive segregants were identified among RILs for all FHB variables except for FHB INC in the MOSN in 2008 due to very high disease pressure (55–100 % INC). Plants inoculated in FHB nurseries, VASN and MOSN, had high infection levels with maximum incidence, severity, or both up to 100 %.

Correlation coefficients of data from three environments including VASN in 2008 and 2009 and VAFLD in 2009 indicated that FHB variables including INC, SEV, IND, and FDK were significantly correlated with each other in both populations except for FDK with SEV in B/M and for SEV with IND in E/MO in 2009 VAFLD (Supplementary Table S2). Grain DON concentration was correlated with INC and FDK in all three environments for both populations except for DON with FDK in B/M in 2009 VAFLD. Plant height was negatively correlated with these five FHB variables in B/M except for DON in the VASN and VAFLD in 2009. Plant height of E/MO RILs was correlated with all five FHB variables in the 2009 VASN, while it was only correlated with INC and FDK in the 2009 VAFLD test. Flowering time was least correlated with other traits, particularly in the B/M population.

### Genetic mapping analysis

For B/M RILs, 589 DArT markers and 71 SSR markers were used to construct the genetic maps. A total of 468 DArT markers including seven from rye (*Secale cereale*), 28 from triticale (×*Triticosecale*), 433 from wheat, and 54 SSR markers were mapped onto chromosomes. They covered all chromosomes except for 3D and 6D. The total genome coverage is 848.6 with 1.6 cM per marker. However, some chromosomes were not covered very well, including chromosomes 1D, 2D, 4D, 5A, 5D, 6A, and 7D. For E/MO, 39 of the 71 new markers were mapped onto chromosomes 2BS, 2D, 3BL, 4BS, 4DS, and 5A in addition to those on maps published by Liu et al. (2007).

### Significant QTL additive effects and their interaction with environments

Just as interactions between line and year were significant for FHB variables, so were the QTL by environment interactions. The main effects of QTL and QTL by environment interactions were estimated by additive (A) and additive by environment (A × E), respectively, in both populations using combined analyses of data across corresponding environments (Table 1).

**Table 1:** The QTL additive effects and additive by environment interactions for Fusarium head blight variables and other traits of recombinant inbred wheat lines of Becker/Massey and Ernie/MO 94-317 based on data from eight environments in Virginia, Missouri, and Kentucky from 2007 to 2010

FHB variables and traits^a^	QTL^b^	Marker interval	Peak position (cM)	Additive^c^	Additive × environment^d^
B/M					
INC (6)	*Qfhs.vt*-*2DS*	*Ppd*-*D1*-*Rht8*	0.0	1.7	−4.0 (VASN 2008), 6.3 (KYFLD 2008)
	*Qfhs.vt*-*4BS*	*Rht*-*B1*-wPt1708	0.0	−1.4	−4.6 (VASN 2008)
GHSEV (2)	*Qfhs.vt*-*1DS*	wPt1595-wPt7946 (1DS-5)^e^	0.8	3.1	−3.1 (VAGH 2007), 3.0 (VAGH 2008)
	*Qfhs.vt*-*3BL*	wPt4048-Xbarc164	24.9	6.2	−5.4 (VAGH 2007), 5.4 (VAGH 2008)
IND (5)	*Qfhs.vt*-*1AS*	wPt4735-wPt3870 (1AS-3)	36.5	1.3	ns^f^
	*Qfhs.vt*-*2BL(2BL*-*6)*	wPt0628-wPt2528	0.9	−0.8	1.6 (VASN 2008), −2.2 (MOSN 2008)
	*Qfhs.vt*-*4BS*	tPt0602-wPt3908	12.2	−1.1	−2.0 (VASN2008), 1.3 (VASN 2007)
	*Qfhs.vt*-*4DS*	*Rht*-*D1*-rPt4471	1.0	1.3	2.0 (VASN2008)
	*Qfhs.vt*-*6BL*	wPt5176-wPt8268	5.1	ns	2.5 (VASN2008)
FDK (3)	*Qfdk.vt*-*4BS*	wPt6149-tPt0602	19.2	−2.7	ns
DON (3)	*Qdon.vt*-*4DL*	wPt3743-wPt6059	34.5	0.4	−0.5 (VASN 2009), 0.8 (VASN 2008)
FT (2)	*Qft.vt*-*4DL*	wPt3743-wPt6059	33.5	1.1	ns
HT (3)	*Qht.vt*-*7A*	wPt5479-rPt6430	18.9	1.8	ns
	*Qht.vt*-*2DS*	Xwmc112-Xwmc503	8.9	−1.9	ns
E/MO					
INC (4)	*Qfhs.umc*-*4BS*	*Rht*-*B1*-Xgwm513	2.0	3.0	4.2 (VASN2008), -2.8 (VAFLD2009),
	*Qfhs.umc*-*4DS*	*Rht*-*D1*	0.0	−4.1	−4.1 (VASN2008), 2.2 (MOSN2008), 3.0 (VAFLD2009)
	*Qfhs.umc*-*5AL*	Xgwm291-*B1*	20.7	−1.9	−2.0 (VASN2008), -1.7 (VASN2009), 2.6 (VAFLD2009)
SEV (4)	*Qfhs.umc*-*2DS*	*Ppd*-*D1*	53.4	−2.7	−3.3 (VASN2008)
	*Qfhs.umc*-*4BS*	*Rht*-*B1*-Xgwm513	6.0	2.2	2.7 (VASN2008), −3.6 (VAFLD2009)
	*Qfhs.umc*-*4DS*	*Rht*-*D1*-Xbarc334b	5.0	−4.2	−1.7 (VASN2008)
	*Qfhs.umc*-*5AL*	Xgwm291-*B1*	20.7	−1.2	−2.4 (VASN2008), 2.6 (VAFLD2009)
GH SEV (2)^g^	*Qfhs.umc*-*3BL*	Xwmc307-Xwmc1	17.2	−6.6	ns
	*Qfhs.umc*-*4BS*	*Rht*-*B1*-Xgwm513	9.0	−8.3	ns
IND (4)	*Qfhs.umc*-*2DS*	*Ppd1*-*D1*	53.4	−1.8	−2.1 (VASN2008)
	*Qfhs.umc*-*4BS*	*Rht*-*B1*-Xgwm513	4.0	3.1	3.6 (VASN2008), -2.5 (VAFLD2009)
	*Qfhs.umc*-*4DS*	*Rht*-*D1*-Xbarc334b	3.0	−4.2	−3.7 (VASN2008), 3.2 (VAFLD2009)
	*Qfhs.umc*-*5AL*	Xgwm291-*B1*	20.7	−1.7	−2.2 (VASN2008), 1.9 (VAFLD2009)
FDK (3)	*Qfdk.umc*-*4BS*	*Rht*-*B1*-Xgwm513	3.0	7.8	ns
	*Qfdk.umc*-*4DS*	*Rht*-*D1*	0.0	−7.5	2.0 (VASN2008)
GH FDK (2)	*Qfdk.umc*-*3BL*	Xwmc653-Xwmc307	13.6	−6.4	ns
	*Qfdk.umc*-*4BS*	*Rht*-*B1*-Xgwm513	6.0	−7.9	ns
DON (3)	*Qdon.umc*-*4DS*	*Rht*-*D1*	0.0	−0.4	0.4 (VASN2008), −0.6 (VASN2009)
	*Qdon.umc*-*6AL*	XE37M59_4-Xbarc171	11.0	−0.3	−0.4 (VASN2009)
GH DON (2)	*Qfhs.don*-*3BL*	Xwmc307-Xwmc1	16.2	−8.0	ns
	*Qfhs.don*-*4BS*	*Rht*-*B1*-Xgwm513	5.0	−7.4	ns
FT (2)	*Qft.umc*-*2AL*	Xgwm122-Xwmc261c	31.2	−0.5	ns
	*Qft.umc*-*2DS*	*Ppd*-*D1*	53.4	1.2	0.6 (VASN2008), −0.7 (VAFLD2009)
HT (3)	*Qht.umc*-*2BL*	Xgwm630a-Xgwm319	12.6	−3.3	ns
	*Qht.umc*-*2DS*	*Ppd1*-*D1*	53.4	2.7	ns
	*Qht.umc*-*4BS*	*Rht*-*B1*-Xgwm513	4.0	−10.0	ns
	*Qht.umc*-*4DS*	*Rht*-*D1*-Xbarc334	4.0	10.8	ns

^a^Abbreviations for FHB variables, traits, and environments: *INC* incidence (%), *SEV* severity (%), *IND* index (0–100), *FDK* Fusarium damaged kernels (%), *DON* deoxynivalenol (mg kg^−1^), *FT* flowering time (d from Jan 1), *HT* height (cm), *B/M* Becker/Massey RILs, *E/MO* Ernie/MO 94-317 RILs, *GH* Greenhouse, number in the parenthesis after trait is the number of unique environments used to identify significant additive and A × E effects

^b^In the QTL name, vt—B/M RIL population was developed at Virginia Tech, umc—E/MO RIL population was developed at University of Missouri–Columbia

^c,d^Significant additive effects and interaction effects between main additive effects and environments estimated using QTLNetwork 2.0 (Yang et al. 2008), negative sign of effects means that the QTL allele from the female parents (Becker or Ernie) decreasing FHB or other traits while the positive sign of effects means that the favorable QTL alleles from male parents (Massey or MO 94-317) to decrease FHB or other traits

^e^The physical bin location of Diversity Array Technology (DArT) markers based on deletion analyses (http://www.cerealsdb.uk.net, accessed on April 15, 2013)

^f^ *ns* not significant at *P* < 0.05

^g^GH SEV, FDK and DON were re-analyzed using QTLNetwork 2.0 based on data collected from point inoculation tests in Columbia, MO, USA (Liu et al. 2007; Abate et al. 2008)

In B/M, ten QTL were associated with GHSEV, INC, SEV, IND, FDK, DON, FT, and HT (Table 1). Among the eight QTL associated with FHB variables, six had favorable effects derived from Massey (positive effects), while only the 4BS and 2BL QTL had favorable effects from Becker. The Becker allele of the 4BS QTL decreased INC, IND, and FDK while the 2BL QTL only decreased IND. The QTL *Qfhs.vt*-*2DS* had a significant additive effect on INC in six environments, which is estimated to be at the same position as *Ppd*-*D1*, at which Massey carries the allele for photoperiod sensitivity (*Ppd*-*D1b*). Two QTL on chromosomes 1DS and 3BL decreased GH SEV while the 1AS QTL decreased IND. The 4DS QTL, with the peak close to *Rht*-*D1b*, reduced IND. Another QTL on chromosome 4DL, 34.5 cM away from *Rth*-*D1b*, reduced DON and FT. Two QTL on chromosomes 7A and 2DS decreased HT. None of the A × E interactions were significant for QTL associated with FT or HT, while all the QTL for FHB variables had significant A × E interactions except for *Qfhs.vt*-*1AS* on IND. The main additive effect of *Qfhs.vt*-*6BL* was not significant.

In E/MO, seven QTL associated with INC, SEV, IND, FDK, DON, FT and HT were identified based on field data from two to four environments in E/MO (Table 1) in addition to the 3BL and 4BS QTL based on GH SEV, FDK, and DON published previously (Liu et al. 2007; Abate et al. 2008). At all QTL, the favorable alleles decreasing FHB variables were from Ernie except the QTL on chromosome 4BS where the favorable allele (*Rht*-*D1a*) was from MO 94-317. The three genes, *Ppd*-*D1*, *Rht*-*B1*, and *Rht*-*D1*, have pleiotropic effects, with the alleles for photoperiod insensitivity or semi-dwarf stature increasing FHB susceptibility. The allele conferring awnless spikes in Ernie for gene *B1* decreased FHB. Independent of these four genes, QTL *Qdon.umc*-*6AL* with favorable allele from Ernie, was associated with lower DON. Two other QTL, *Qft.umc*-*2AL* and *Qht.umc*-*2BL*, decreased FT and HT with alleles from Ernie. Allele *Ppd*-*D1a* from MO 94-317 decreased both FT and HT and *Rht*-*B1b* and *Rht*-*D1b* reduced height.

A × E interactions in E/MO were significant for all four genes (*Ppd*-*D1*, *Rht*-*B1*, *Rht*-*D1*, and *B1*) associated with FHB variables except for *Rht*-*B1b* on FDK and GH SEV, GH FDK, and GH DON. Only *Ppd*-*D1* with FT had a significant A × E interaction among the five QTL for FT and HT.

### Epistasis and interaction effects between epistasis and environments based on two-locus analyses

In B/M, four pairs of QTL have significant A × A epistases for INC, GH SEV, FDK, and HT but only the pair between *Qfhs.vt*-*4DS* and *Qfhs.vt*-*6AS* had significant A × A × E interactions (Table 2). Two other pairs of QTL only had significant A × A × E interactions for INC or IND. Only the pair of QTL for IND, *Qfhs.vt*-*1AS* and *Qfhs.vt*-*6BL*, were from two QTL with significant additive effects or A × E interactions (Table 1).

**Table 2:** Epistasis and interaction effects between epistasis and environment of Fusarium head blight variables and other traits for recombinant inbred wheat lines of Becker/Massey and Ernie/MO 94-317 based on data from eight environments at Virginia, Missouri, and Kentucky from 2007 to 2010

FHB variables and traits^a^	QTL^b^	Marker interval	Peak position (cM)	QTL	Marker interval	Peak position (cM)	Additive × additive	Additive × additive × environment^c^
B/M								
INC	*Qfhs.vt*-*2A*	wPt-6711-wPt-9320	27.4	*Qfhs.vt*-*6AS* (6AS-1or 5)^e^	wPt-1664-wPt-9687	3.1	ns^d^	−2.5 (VASN 2009), 4.3 (VASN 2007), −3.6 (KYFLD 2008)
	*Qfhs.vt*-*4DS*	Xcfd71b-wPt-3743	25.8	*Qfhs.vt*-*6AS* (6AS-1or 5)	wPt-8539-wPt-0959	15.2	2.0	3.5 (VASN2008), 4.2 (VASN2007), −4.2 (KYFLD 2008)
GH SEV	*Qfhs.vt*-*4BS*	Xwmc48b-wPt-6149	6.7	*Qfhs.vt*-*5B3*	wPt-7240-wPt-0819	0	−3.0	ns
IND	*Qfhs.vt*-*1AS*	wPt-4735-wPt-3870	36.5	*Qfhs.vt*-*6BL*	wPt-5176-wPt-8268	5.1	ns	1.3 (VASN 2008)
FDK	*Qfdk.vt*-*1AL(1AL*-*3)*	wPt-6005-wPt-1310	0.2	*Qfdk.vt*-*2BL*	wPt-2186-wPt-2314	6.3	4.0	ns
HT	*Qht.vt*-*2A*	wPt-3114-tPt-8937	61.6	*Qht.vt*-*3A*	wPt-2127-wPt-3697	26.2	2.0	ns
E/MO								
SEV	*Qfhs.umc*-*2DS*	*Ppd*-*D1*	53.4	*Qfhs.umc*-*4DS*	*Rht*-*D1*-Xbarc334	5.0	1.7	ns
	*Qfhs.umc*-*4BS*	*Rht*-*B1*-Xgwm513	6.0	*Qfhs.umc*-*4DS*	*Rht*-*D1*-Xbarc334	5.0	ns	4.1 (VASN2008), −2.2 (MOSN2008), −2.2 (VASN2009)
GH SEV^f^	*Qfhs.umc*-*3BL*	Xwmc307-Xwmc1	17.2	*Qfhs.umc*-*5AS*	Xwmc446-Xbarc56	17	−4.2	ns
IND	*Qfhs.umc*-*2DS*	*Ppd*-*D1*	53.4	*Qfhs.umc*-*4DS*	*Rht*-*D1*-Xbarc334	3.0	1.2	ns
	*Qfhs.umc*-*4BS*	*Rht*-*B1*-Xgwm513	4.0	*Qfhs.umc*-*4DS*	*Rht*-*D1*-Xbarc334	3.0	ns	2.0 (VASN2008), −1.5 (MOSN2008)
FDK	*Qfdk.umc*-*4BS*	*Rht*-*B1*-Xgwm513	3.0	*Qfdk.umc*-*4DS*	*Rht*-*D1*	0.0	ns	2.5 (VASN2008), −3.4 (VAFLD2009)
DON	*Qdon.umc*-*6AL*	XE37M59_4-Xbarc171	11.0	*Qdon.umc*-*4DS*	*Rht*-*D1*	0.0	0.2	ns
GH DON^g^	*Qdon.umc*-*3BL*	Xwmc307-Xwmc1	16.2	*Qdon.umc*-*5AS*	Xwmc446-Xbarc56	16	−4.6	ns
FT	*Qft.umc*-*2AL*	Xgwm122-Xwmc261c	31.2	*Qft.umc*-*2DS*	*Ppd*-*D1*	53.4	−0.5	ns
HT	*Qht.umc*-*4BS*	*Rht*-*B1*-Xgwm513	4.0	*Qht.umc*-*4DS*	*Rht*-*D1*-Xbarc334	4.0	4.1	ns

^a^Abbreviations for FHB variables, traits, and populations: *INC* incidence (%), *SEV* severity (%), *IND* index (0–100), *FDK* *Fusarium* damaged kernels (%), *DON* deoxynivalenol (mg kg^−1^), *FT* flowering time (days from Jan 1), *HT* height (cm), *B/M* Becker/Massey RILs, *E/MO* Ernie/MO 94-317 RILs

^b^In QTL name, vt—B/M RIL population was developed at Virginia Tech, umc—E/MO RIL population was developed at University of Missouri–Columbia

^c^Interaction effects between additive by additive and environments, VASN 2008 and 2009—Virginia scab nursery in 2008 and 2009, VAFLD 2009—Virginia field under natural infection in 2009, MOSN 2008—Scab nursery at Columbia, Missouri in 2008

^d^ *ns* not significant at *P* < 0.05

^e^The physical bin location of Diversity Array Technology (DArT) markers based on deletion analyses (http://www.cerealsdb.uk.net, accessed on April 15, 2013)

^f,g^Based on data from greenhouse point inoculation at Columbia, MO, USA in 2002 and 2003 (Liu et al. 2007; Abate et al. 2008)

In E/MO, *Qfhs.umc*-*4DS* (close to *Rht*-*D1*) was involved in seven of the ten A × A epistatic interactions. It had significant effects with *Ppd*-*D1* for SEV and IND, with *Qdon.umc*-*6AL* for DON, and with the *4BS QTL* for SEV, IND, FDK, and HT. In addition, there were significant A × A interactions between the 5AS and 3BL QTL for GH SEV and GH DON with favorable alleles from Ernie. The A × A interactions between *Ppd*-*D1* and *Qft.umc*-*2AL* shortened FT with alleles from Ernie. Significant A × A × E interactions only existed between the two QTL on chromosomes 4BS (*Rht*-*B1b*) and 4DS (*Rht*-*D1b*) in E/MO for SEV, IND and FDK. The QTL on chromosome 4DS was involved in most of the epistatic interactions and in all of the A × A × E interactions.

### Comparisons of FHB resistance among RIL groups with different allelic combinations of genes

A set of 115 B/M RILs with different allelic combinations of *Rht*-*B1b*, *Rht*-*D1b*, *Rht8c,* and *Ppd*-*D1a* was analyzed using the group means of all traits including FT, HT and FHB variables from VASN in 2008 and 2009 (Table 3). The few double dwarf (*Rht*-*B1b* and *Rht*-*D1b*) RILs were excluded in the mean comparisons as their growth was abnormal in general. RILs in group 7 (*Rht*-*D1b*, *Ppd*-*D1a*) had higher INC and IND, and those in group 6 (*Rht*-*B1b*, *Ppd*-*D1b*) had higher FDK than those in group 9 (*Rht8c*, *Ppd*-*D1a*). There were no significant differences among RIL groups for SEV, DON, FT, HT, or GHSEV, which may have been the result of the relatively small sample size within some groups. When alleles of other genes are same, the *Ppd*-*D1a* group was 1.1–3.5 days earlier in FT and 4.4–13.3 cm shorter than those of the *Ppd*-*D1b* groups except for HT in groups 9 and 10 (Table 3).

**Table 3:** Means of Fusarium head blight variables and traits of recombinant inbred line (RIL) groups having different allelic combinations of gene alleles *Rht*-*B1b*, *Rht*-*D1b*, *Rht8c* and *Ppd*-*D1a* in Becker/Massey wheat population from 2008 to 2009 Virginia scab nursery and greenhouse severity in 2008

RIL groups	Allelic combinations	No. of RILs	INC^a^	SEV	IND	FDK	DON	FT	HT^b^	GHSEV^c^
1	*Rht*-*B1b, Rht8c, Ppd*-*D1a*	16	47.9ab	25.8a	15.2ac	42.6ac	4.7a	134.1a	101.8a	56.2a
2	*Rht*-*B1b, Rht8c, Ppd*-*D1b*	4	44.4ab	19.5a	10.8ab	39.2ab	5.9a	137.6a	111.9a	41.9a
3	*Rht*-*D1b, Rht8c, Ppd*-*D1a*	15	50.9a	23.3a	14.7ab	40.3ab	4.6a	135.6a	104.4a	66.1a
4	*Rht*-*D1b, Rht8c, Ppd*-*D1b*	3	44.6ab	23.3a	13.9ab	41.3ab	5.8a	136.9a	110.0a	74.2a
5	*Rht*-*B1b, Ppd*-*D1a*	5	43.0ab	25.8a	13.2ab	43.9ab	3.2a	134.3a	109.3a	62.0a
6	*Rht*-*B1b, Ppd*-*D1b*	15	47.0ab	22.4a	12.4ab	45.2a	4.9a	137.5a	113.7a	56.2a
7	*Rht*-*D1b, Ppd*-*D1a*	8	54.4a	25.1a	17.0a	43.2ab	4.0a	135.7a	103.5a	62.9a
8	*Rht*-*D1b, Ppd*-*D1b*	5	42.3ab	18.5a	9.7ab	41.9ab	5.9a	138.9a	116.8a	60.9a
9	*Rht8c, Ppd*-*D1a*	18	37.1b	20.2a	8.5b	28.9b	3.5a	134.7a	108.7a	70.5a
10	*Rht8c, Ppd*-*D1b*	11	39.0ab	20.7a	9.6ab	28.4bc	4.5a	135.8a	107.1a	59.1a
11	*Ppd*-*D1a*	3	33.0ab	17.8a	6.3abc	29.3ab	3.9a	136.3a	103.0a	55.6a
12	Ppd-D1b	12	39.2ab	19.1a	8.4bc	34.6ab	4.2a	137.4a	113.9a	70.1a
	HSD^e^		21.5	9.9	10.1	21.2	4.2	7.7	24.3	41.7
	Minimum HSD		13.2	6.1	6.2	13.0	2.6	4.7	15.0	25.7

^a^Abbreviations of FHB variables and traits: *INC* incidence (%), *SEV* severity (%), *IND* index (0–100), *FDK* *Fusarium* damaged kernels (%), *DON* deoxynivalenol (mg kg^−1^), *FT* flowering time (days from Jan 1), *HT* height (cm), *GH* greenhouse

^b^HT (height) was only measured from VASN in 2009

^c^Severity from greenhouse based on point inoculation. B/M RILs were screened in VAGH in 2008

^d^Means followed by the same letter within a column are not significantly different at *P* < 0.05. The significance was set based on the SAS output of pairwise comparisons converted into letters

^e^Tukey’s studentized range (HSD) values were calculated based on Harmonic means of number of lines in all 12 groups; minimum HSD values were calculated by using the harmonic means of two groups with the largest number of lines

In the E/MO population, 12 groups of RILs composed of various allelic combinations of four genes including *B1*, *Rht*-*B1b*, *Rht*-*D1b*, and *Ppd*-*D1a* were analyzed for differences among FHB variable and trait means (Table 4). Among 191 RILs, group 7 (*Rht*-*D1b*, *Ppd*-*D1a*) had the earliest FT and highest INC, SEV, and IND. The RILs in groups 1–8 were shorter in HT and had high values for INC, SEV, IND, FDK, and DON. As in the B/M population, comparisons among E/MO RIL groups differing only for *Ppd*-*D1* alleles indicate that groups having the *Ppd*-*D1a* allele tended to flower earlier (1.6–6.1 days) and were shorter in height (2.2–6.9 cm) than those with *Ppd*-*D1b*. Groups of RILs with one dwarfing gene had intermediate HT ranging from 90.7 to 100.3 cm while those without dwarf genes ranged from 105.3 to 112.1 cm. In both populations, RILs with semi-dwarfing genes *Rht*-*B1b* or *Rht*-*D1b* tended to have higher values for FHB variables than RILs lacking dwarfing genes.

**Table 4:** Means of Fusarium head blight variables and traits of recombinant inbred line (RIL) groups having different allelic combinations of gene alleles *B1*, *Rht*-*B1b*, *Rht*-*D1b*, and *Ppd*-*D1a* in Ernie/MO 94-317 wheat population from 2008 to 2009 scab nursery and greenhouse severity in 2002 and 2003

RIL groups	Allelic combinations	No. of RILs	INC^a^	SEV	IND	FDK	DON	FT	HT^b^	GHSEV^c^
1	*B1, Rht*-*B1b, Ppd*-*D1a*	15	45.9abc	31.5bde	18.3bd	42.6ac	2.3a	134.8cd	96.9de	63.9ab
2	*B1, Rht*-*B1b, Ppd*-*D1b*	13	40.0bde	23.3cde	12.1cde	49.1a	3.0a	138.9ab	99.2b-e	56.1ab
3	*B1, Rht*-*D1b, Ppd*-*D1a*	12	46.0a-d	32.7abd	18.7bd	48.0ac	3.1a	134.4cd	90.7e	75.4a
4	*B1, Rht*-*D1b, Ppd*-*D1b*	6	44.4a-e	21.4bce	15.1bde	51.1ac	3.4a	140.6a	93.8de	57.6ab
5	*Rht*-*B1b, Ppd*-*D1a*	9	49.9abc	36.9ab	23.8ab	36.6abc	2.3a	134.8bcd	93.7de	43.6b
6	*Rht*-*B1b, Ppd*-*D1b*	16	48.8abc	30.7bde	19.2bc	40.1ac	3.1a	137.8abc	100.3a-e	55.8ab
7	*Rht*-*D1b, Ppd*-*D1a*	12	56.5a	43.6a	28.9a	32.9bcd	3.3a	133.6d	96.3de	69.2ab
8	*Rht*-*D1b, Ppd*-*D1b*	18	51.5ab	27.0b-e	17.0bd	39.4ac	3.6a	139.1a	100.0b-e	59.3ab
9	*B1, Ppd*-*D1a*	22	33.0de	21.9ce	9.3e	22.9bd	1.8a	134.3d	107.7a-d	69.5a
10	*B1, Ppd*-*D1b*	32	32.0e	20.2ce	7.7e	25.3bd	2.4a	137.1abc	109.9ab	64.4ab
11	*Ppd*-*D1a*	18	38.5cde	26.4ce	13.1cde	22.5bd	2.6a	134.8cd	105.3a-d	65.3ab
12	*Ppd*-*D1b*	18	36.6cde	19.8ce	9.7de	20.3d	2.3a	136.6a-d	112.1a	62.9ab
	HSD^e^		14.8	11.3	9.6	14.7	2.3	3.8	13.7	24.5
	Minimum HSD		10.6	8.1	6.9	10.6	1.7	2.7	9.8	17.6

^a^Abbreviations of traits: *INC* incidence (%), *SEV* severity (%), *IND* index (0–100), *FDK* Fusarium damaged kernels (%), *DON* deoxynivalenol (mg kg^−1^), *FT* flowering time (days from Jan 1), *HT* height (cm), *GH* greenhouse

^b^HT (height) was only measured from VASN in 2009

^c^Severity from greenhouse based on point inoculation. E/MO RILs were screened at Columbia, MO in 2002 and 2003 (Liu et al. 2007; Abate et al. 2008)

^d^Means followed by the same letter within a column are not significantly different at *P* < 0.05. The significance was set based on the SAS output of pairwise comparisons converted into letters. “-”between letters represents those letters omitted between these two border letters

^e^Tukey’s studentized range (HSD) values were calculated based on Harmonic means of number of lines in all 12 groups; Minimum HSD values were calculated by using the harmonic means of two groups with the largest number of lines

### Parental alleles of linked markers for common QTL

Tightly linked markers and base pair sizes of parental alleles for genes and QTL associated with FHB resistance are presented for the B/M and E/MO populations (Supplementary Table S3). These markers have been used to screen elite breeding lines from >10 SRW wheat breeding programs by the USDA-ARS Genotyping Center at Raleigh, NC, USA (http://www.ars.usda.gov/Main/docs.htm?docid=19523&page=4). The two regional experiments are Northern Uniform Winter Wheat Scab Nursery (NUWWSN, 60 entries) and Uniform Southern Fusarium Head Blight Nursery (USFHBN, 51 entries). A set of 43 (72 %) and 22 (43 %) lines had *Ppd*-*D1b* while only a few had *Rht8c*. About 5–30 % may have the 3BL QTL conditioning GH SEV resistance. About 45–70 % of the tested lines have alternative wild alleles of *Rht*-*B1* or *Rht*-*D1* to reduce the values of FHB variables.

## Discussion

### Correlation among FHB variables and other morphological traits

The FHB variables INC, SEV, IND, and FDK were highly and significantly correlated with each other in both populations in diverse environments (Supplementary Table S2). Correlations of DON concentration with INC and FDK also were significant in most environments, while DON was not significantly associated with SEV in most of the tests. This suggests that DON concentration in most environments is determined to a larger extent by FHB incidence (percentage of infected spikes) than severity (percentage of diseased spikelets per spike). The concentration of DON in grain also is affected by the type and prevalence of *F.* *graminearum* isolates (3ADON, 15ADON) and by the prevailing environmental conditions from spike emergence to grain harvest (Cowger et al. 2009). FT and HD were positively related with DON at VAFLD in 2009, which might indicate that genotypes having early heading and flowering times escaped the optimal infection conditions, thus resulting in lower DON.

### Comparison of mapped QTL to previously known QTL

The chromosome locations of QTL mapped in this study were compared with previously mapped QTL based on two wheat consensus maps, one meta-analysis QTL map, and two DArT marker maps. The two wheat consensus maps are integrated maps (Somers et al. 2004) and the International *Triticeae* Mapping Initiative maps (ITMI) (Song et al. 2005) containing GWM, WMC, and BARC SSR markers with bin maps confirmed are from Sourdille et al. (2004). The meta-analysis map of QTL for FHB resistance includes most mapped QTL from sources worldwide reported from 2001 to 2009 (Liu et al. 2009). The two DArT marker maps integrated DArT and SSR markers using double haploid populations derived from Cranbrook to Halberd (Kammholz et al. 2001) and from Arina to NK93604 (Semagn et al. 2006, 2007). If the DArT markers are absent on these two maps, DArT markers and their genetic and physical chromosome locations were referenced from the Triticarte (http://www.triticarte.com.au/content/further_development.html, accessed on April 1, 2013) and cereals DB (Wilkinson et al. 2012, www.cerealsdb.uk.net, accessed on April 15, 2013) websites.

**Fig. 1:**
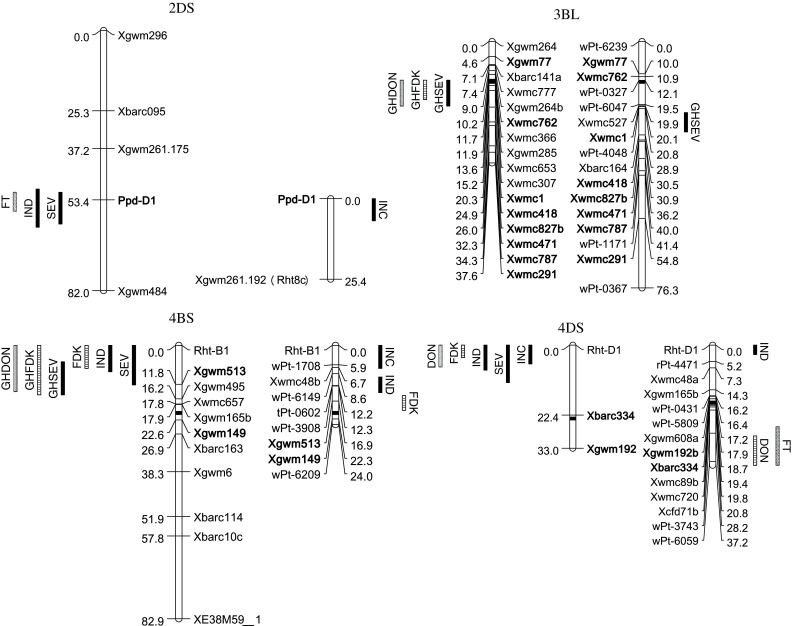
Four common quantitative trait loci (QTL) associated with Fusarium head blight (FHB) variables and other traits in Ernie/MO 94-317 (E/MO, *left*) and Becker/Massey (B/M, *right*) populations. For each pair of chromosomes and QTL *bars*, the one on the left is for E/MO and the one on the *right* is for B/M. Markers in common on chromosomes in both populations are in *bold font*. The *bar length* is the range of the QTL significant regions (*P* < 0.05) from the output of QTLNetwork 2.0 (Yang et al. 2008). The label for each QTL of FHB variables besides the *bar* is: *INC* incidence (%), *SEV* severity (%), *IND* index (0–100), *FDK* Fusarium damaged kernels (%), *DON* deoxynivalenol (mg kg^−1^), *FT* flowering time (days from Jan 1), *HT* height (cm), ‘GH’ in front of FHB variables means the data from point inoculation in the greenhouse, B/M data were from the greenhouse at Blacksburg, Virginia, in 2007 and 2008 while E/MO data were from the greenhouse at Columbia, Missouri, in 2002 and 2003 (Liu et al. 2007; Abate et al. 2008). The genetic maps and graphs were drawn using MapChart 2.0 (Voorrips 2002)

All four significant QTL common in B/M and E/MO populations on chromosomes 2DS, 3BL, 4BS, and 4DS can be aligned based on linked SSR markers on consensus maps and the meta-analysis QTL map (Fig. 1). In addition, *Qfhs.umc*-*5AL* overlapped the *B1* gene in E/MO and awnless plants had lower FHB, which is consistent with what Gervais et al. (2003) found in the European cultivar Renan. Other QTL, *Qfhs.vt*-*1AS*, *Qfhs.vt*-*1DS*, *Qfhs.vt*-*2BL*, *Qdon.vt*-*4DL* in B/M and *Qdon.umc*-*6AL* in E/MO, are independent of the morphological genes evaluated in the current study.

In B/M, DArT marker wPt-3870 linked to *Qfhs.vt*-*1AS* was located at bin 1AS-3 where Xgwm33 and Xwmc818 are located. Marker Xwmc818 was reported to be linked to QTL for SEV in Pirat (*Rht*-*D1b*) (Holzapfel et al. 2008). A QTL for DON accumulation was located in the same region of Wheaton (Yu et al. 2008; Liu et al. 2009). The marker wPt-7946 linked to *Qfhs.vt*-*1DS* is very close to wPt-3738 which is in bin 1DS-5. Marker Xbarc149 in this region was reported to be linked to a QTL from Pirat (Holzapfel et al. 2008). The DArT marker wPt-0628 linked to *Qfhs.vt*-*2BL* was located in bin 2BL-6 where Xgwm501 and Xgwm47 are located. These two markers were associated with lower SEV in Ning 7840, SD97060, and G16-92 (Zhou et al. 2002; Malla et al. 2010; Schmolke et al. 2008). The DArT marker wPt-3132 linked to the 2BL QTL in the current study also was linked to the QTL in SD97060. Markers wPt-5809 and wPt-0431 are 20 cM away from wPt-3743 to wPt-6509, which places the latter on chromosome 4DL region close to markers Xwmc331 and Xwmc457 where a meta-QTL from DH181 for INC and FDK, and from Arina for FDK was present (Yang et al. 2005; Draeger et al. 2007; Liu et al. 2009).

The QTL *Qdon.umc*-*6AL* from E/MO, linked to marker Xbarc171, was close to Xbarc107 that was associated with type II resistance in Apache, Dream, and Spark (Holzapfel et al. 2008; Schmolke et al. 2005; Srinivasachary et al. 2008).

*Qfhs.vt*-*3BL* and *Qfhs.umc*-*3BL* are in the same region as the QTL identified in the Swiss winter wheat cultivar Arina (Paillard et al. 2004), French cultivar Apache (Holzapfel et al. 2008) and Chinese landrace Wangshuibai (Zhou et al. 2004; Yu et al. 2008) which were associated with reduced FHB severity in greenhouse and/or field experiments. This QTL was placed near the centromere of chromosome 3BS based on markers Xgwm77, Xgwm285, and Xgwm376 on the ITMI map (Song et al. 2005). Results from the current study including additional SSR markers, Xwmc1, Xwmc418, and Xwmc827, indicated that this QTL likely is located on 3BL, which is consistent with the integrated map of Somers et al. (2004). This 3BL QTL is the only common QTL associated with greenhouse SEV in both populations with additional effects on reducing FDK and DON by alleles from Ernie (Liu et al. 2007; Abate et al. 2008) (Table 1; Fig. 1).

The QTL on chromosome 2DS is in the same region as QTL associated with FHB INC, SEV, and DON in Sumai 3 (Handa et al. 2008), SEV in cultivars Biscay and Romanus (Holzapfel et al. 2008), and Chinese landrace Wangshuibai (Jia et al. 2005) based on the chromosome locations of markers Xgwm261 and Xgwm484 (Fig. 1). This QTL was located at the *Ppd*-*D1* locus in both populations in this study; however, *Ppd*-*D1* was not mapped in those three above-mentioned studies (Table 1; Fig. 1). In most environments, *Ppd*-*D1a* is associated with early head emergence and shorter plant height. Since infection of wheat heads occurs near flowering time, differences among genotypes in flowering date and plant height and environmental conditions during this period can potentially influence initial infection and disease development. In the current study, spray inoculations were conducted according to the flowering time to minimize the effects of different flowering time and height on FHB infection. In E/MO, the photoperiod-sensitive allele, *Ppd*-*D1b* from Ernie, was associated with lower SEV and IND, later flowering time, and taller plant height, while the *Ppd*-*D1b* from Massey only decreased INC in B/M among the six environments (Table 1). The *Ppd*-*D1a* allele had smaller effects on reducing HT and increasing FHB variables when compared with those of *Rht*-*B1b* and *Rht*-*D1b* based on the magnitude of additive effects and group means with only alleles of *Rht*-*B1b*, *Rht*-*D1b*, and *Ppd*-*D1a*, especially in E/MO (Tables 1, 3, 4).

The QTL on chromosome 4BS in both populations are in a region overlapping gene *Rht*-*B1* where QTL have been identified for type I resistance (FHB incidence) in Wuhan 1 (Somers et al. 2003) and Wangshuibai (Lin et al. 2006), type II resistance (FHB severity) in greenhouse studies in Ernie (Liu et al. 2007) and Wangshuibai (Jia et al. 2005), and resistance to DON accumulation and kernel damage (FDK) in two SRW wheat genotypes Ernie (Abate et al. 2008) and IL94-1653 (Bonin and Kolb 2009) based on markers Xgwm513, Xgwm495, and Xgwm149 (Somers et al. 2004). However, in the current study, the favorable QTL alleles for all the FHB variables except GHSEV, GH FDK, and GH DON were derived from the susceptible parents, Becker or MO 94-317 that have the wild-type allele *Rht*-*B1a* (Table 1). In E/MO, the 4BS QTL decreased INC, SEV, IND, and FDK based on data field spray inoculation.

The QTL on chromosome 4DS are in a region close to gene *Rht*-*D1* where major QTL have been previously identified and associated with FHB incidence and severity in the cultivars Soissons and Spark (Srinivasachary et al. 2009, 2008), Apache, History, Romanus (Holzapfel et al. 2008), and Arina (Draeger et al. 2007) based on the chromosome locations of *Rht*-*D1* and SSR markers Xbarc334 and Xgwm192. Similar to that of Ernie and Massey, these six European wheat cultivars have *Rht*-*D1a* which provided the resistance to FHB. The *Qdon.vt*-*4DL* from Massey to *Qdon.umc*-*4DS* from Ernie co-localized with two meta-QTL on chromosome 4DL and 4DS, respectively (Liu et al. 2009). QTL *Qdon.vt*-*4DL* on 4DL for DON and *Qfdk.umc*-*4DS* on 4DS for FDK have not been reported previously in other sources.

In E/MO, the semi-dwarfing alleles *Rht*-*B1b* and *Rht*-*D1b* on chromosomes 4BS and 4DS decreased plant height by 10.0–10.8 cm (Table 1). The pleiotropic effects of *Rht*-*B1b* and *Rht*-*D1b* observed in E/MO and B/M are consistent with previous reports of association between these two dwarfing genes with FHB susceptibility in European wheat cultivars (Srinivasachary et al. 2008; Hilton et al. 1999; Draeger et al. 2007). In tests conducted in Germany (Knopf et al. 2008) and the UK (Gosman et al. 2007) that included wheat cultivars with dwarfing gene allele *Rht*-*B1b* or *Rht*-*D1b*, cultivars having *Rht*-*D1b* were more susceptible to FHB and had higher disease incidence than standard height cultivars (*Rht*-*D1a*). Srinivasachary et al. (2009) also reported that both *Rht*-*B1b* and *Rht*-*D1b* decrease FHB type I resistance, yet *Rht*-*B1b* could significantly increase type II resistance based on point inoculation of near-isogenic lines which were derived from cultivars Mercia and Maris Huntsman. Similar results were observed in the current study for both cultivars, Ernie and Massey, in greenhouse point inoculation studies.

### Effects of QTL, dwarfing genes, and *Ppd*-*D1* on FHB resistance and impacts on breeding

The 3BL QTL has been identified in cultivars and diverse germplasm in Europe (Paillard et al. 2004; Holzapfel et al. 2008), China (Zhou et al. 2004; Yu et al. 2008), and the US (Liu et al. 2007; Abate et al. 2008). Therefore, availability of tightly linked markers, such as those identified in the current study (Supplementary Table S3), will facilitate marker-assisted selection of this QTL in existing breeding populations to develop cultivars with Type II resistance, lower in FDK and DON (Abate et al. 2008). Success in combining the 3BL QTL with gene *Fhb1*, located on 3BS, will depend on the degree of linkage and whether current lines having both of these in coupling exist.

The *Ppd*-*D1*, *Rht*-*B1*, and *Rht*-*D1* genes have pleiotropic effects on HT and FHB variables. The alleles *Ppd*-*D1b*, *Rht*-*B1a*, and *Rht*-*D1a* reduce FHB and increase HT (Table 1). The *B1* gene from Ernie was associated with lower FHB INC and SEV (Table 1). Lines in both populations lacking either dwarfing gene, *Rht*-*B1b* or *Rht*-*D1b*, have lower values for FHB variables, such as group 8 (*Rht8c*, *Ppd*-*D1a*) (Table 3, 4); therefore, use of *Rht8c* and other dwarfing genes with a similar mode of action likely would be beneficial in breeding programs where FHB is a major priority. However, significant variation for FHB resistance exists among wheat genotypes with *Rht*-*B1b* or *Rht*-*D1b*, thus it should be feasible to select high yielding semi-dwarf lines having moderate-to-high levels of FHB resistance (Voss et al. 2008). This is consistent with results from previous studies involving significant epistasis between *Rht*-*D1b* and *Ppd*-*D1a* (or *Qdon.umc*-*6AL*) with FHB wherein it was postulated that wheat breeders can find germplasm lines containing various combinations of these genes and resistance QTL to minimize pleiotropic effects of these genes on FHB susceptibility. While many US soft red winter cultivars with *Rht*-*D1b* are susceptible to FHB, a few cultivars having *Rht*-*D1b*, such as Roane (Griffey et al. 2001), Tribute (Griffey et al. 2005), and Jamestown (Griffey et al. 2010), express moderate levels of FHB resistance similar to that of Ernie with *Rht*-*B1b* (McKendry et al. 1995; Liu et al. 2005, 2007, 2009).

Among 13 QTL for FHB variables identified in the two populations, only *Qfhs.vt*-*1AS* did not have significant A × E interactions; however, it has significant A × A × E interactions. In E/MO, the A × A interactions of *Rht*-*B1b* and *Rht*-*D1b* were not significant for SEV, IND, and FDK, while their corresponding A × A × E interactions were significant (Table 2). These interactions indicated the complication of FHB resistance. The model used to estimate both main effects and interaction effects of QTL and QTL × E provides an unbiased estimate of QTL main effects and a better understanding of a complex trait, like FHB resistance (Kumar et al. 2007).

In summary, this study characterized FHB resistance in two US soft red winter wheat cultivars. Four significant common QTL associated with INC, SEV, IND, FDK and DON were identified, and all of them overlapped other meta-QTL from FHB-resistant sources worldwide based on common linked molecular markers. Three of them overlapped genes governing plant height (*Rht*-*B1* and *Rht*-*D1*) and photoperiod sensitivity (*Ppd*-*D1*). The pleiotropic effects of *Rht*-*B1b* and *Rht*-*D1b* in E/MO and B/M are consistent with results from previous studies of European wheat cultivars (Srinivasachary et al. 2008; Hilton et al. 1999; Draeger et al. 2007). The current study evaluated the direct association of these genes with FHB resistance in two US wheat cultivars. Results suggest that incorporating and pyramiding FHB resistance QTL using wheat genotypes having desirable morphological genes, such as *Ppd*-*D1a* and *Rht*-*B1b*, can be an effective strategy to improve FHB resistance using marker-assisted selection.

## Electronic supplementary material

Below is the link to the electronic supplementary material.

Supplementary material 1 (DOC 102 kb)
