# Supplementary material for: Molecular characterization of field resistance to Fusarium head blight in two US soft red winter wheat cultivars
Source: Theor Appl Genet. 2013 Jul 6;126(10):2485–98. doi: 10.1007/s00122-013-2149-y (PMC3782633; doi:10.1007/s00122-013-2149-y)
Supplement: Supplementary file 1 — Supplementary material 1 (DOC 102 kb) [file 122_2013_2149_MOESM1_ESM.doc]

**Molecular characterization of field resistance to Fusarium head blight in two U.S. soft red winter wheat cultivars**

Shuyu Liu^1a*^, Carl A. Griffey^1*^, Marla D. Hall^1b^, Anne L. McKendry^2^, Jianli Chen^1c^, Wynse S. Brooks^1^, Gina Brown-Guedira^3^, David Van Sanford^4^, and David G. Schmale^5^

^1^Dept. of Crop and Soil Environmental Sciences, Virginia Tech, Blacksburg, VA 24060

^2^ Dept of Plant Science, University of Missouri, Columbia, MO 65201

^3^ Eastern Regional Small Grains Genotyping Lab, USDA-ARS, Raleigh, NC 27695

^4^Dept. of Plant and Soil Sciences, University of Kentucky, Lexington, KY 40546

^5^Dept. of Plant Pathology, Physiology and Weed Science, Virginia Tech, Blacksburg, VA 24060

^a^Present Address: Texas A&M AgriLife Research and Extension Center, Texas A&M University System, 6500 Amarillo Blvd W., Amarillo, TX, USA 79106

^b^Present Address: Limagrain Cereal Seeds, 6414 N. Sheridan, Wichita, KS 67204, USA

^c^Present Address: Dept of Agronomy, University of Idaho Aberdeen Research & Extension Center, Aberdeen, ID, 83210, USA

*Corresponding Authors: [SLiu@ag.tamu.edu](mailto:SLiu@ag.tamu.edu), [CGriffey@vt.edu](mailto:CGriffey@vt.edu).

Supplementary Table S1 Mean squares of Fusarium head blight variables and other traits of two wheat populations evaluated in field scab nurseries at Blacksburg, Virginia in 2008 and 2009

| Sources | | DF | INC^a^ | SEV | IND | FDK | DON | FT | HT^b^ |
| --- | --- | --- | --- | --- | --- | --- | --- | --- | --- |
| B/M^c^ | | |  |  |  |  |  |  |  |
| Line | 151 | | 623***^d^ | 148*** | 173*** | 571*** | 22 | 57*** | 364*** |
| Line*year | 151 | | 633*** | 154*** | 175*** | 523*** | 24 | 55*** | - |
| Year^e^ | 1 | | 170661* | 28670 | 30838* | 1505 | 227 | 2673*** | - |
| Rep(year) | 2 | | 2558 | 2282 | 1432 | 145 | 272 | 3 | 116* |
| Error | 302 | | 242 | 86 | 76 | 48 | 20 | 11 | 29 |
| E/MO |  | |  |  |  |  |  |  |  |
| Line | 230 | | 902*** | 554*** | 487*** | 1021*** | 14*** | 44*** | 468*** |
| Line*Year | 230 | | 487*** | 311*** | 256*** | 268*** | 10** | 5 | - |
| Year | 1 | | 232925* | 133021** | 101497* | 9870* | 954 | 2237* | - |
| Rep(year) | 2 | | 2695 | 966 | 1315 | 519 | 82 | 41 | 104 |
| Error | 460 | | 265 | 130 | 97 | 40 | 7 | 4 | 66 |

^a^ Abbreviations of traits: INC – Incidence (%), SEV – Severity (%), IND – Index (0-100), FDK – Fusarium damaged kernels (%), DON – Deoxynivalenol (mg kg^-1^), FT – Flowering time (d from Jan 1), HT – Height (cm)

^b^ HT was only recorded at VASN in 2009

^c^ B/M – F_7:14_ RIL from the cross of Becker/Massey, E/MO – F_11_ RILs from the cross of Ernie/MO 94-317

^d^ *, **, *** mean significant at P < 0.05, 0.01, and 0.001

^e^ Year was tested against the term of Rep (year)

Supplementary Table S2 Pearson correlation of means of Fusarium head blight variables and other traits measured in scab nurseries and under natural field conditions in 2008 and 2009 (top-right diagonal data with underline are for E/MO wheat population and bottom-left diagonal are for B/M wheat population)

| Traits (B/M)^a^ | INC^b^ | SEV | IND | FDK | DON | FT (HD) ^c^ | HT^d^ | Traits (E/MO) |
| --- | --- | --- | --- | --- | --- | --- | --- | --- |
| VASN 2008^e^ | |  |  |  |  |  |  |  |
| SEV | 0.53***^e^ | 0.59*** | 0.80*** | 0.52*** | 0.22** | 0.27*** | - | INC |
| IND | 0.82*** | 0.88*** | 0.93*** | 0.42*** | -0.10 | -0.31*** | - | SEV |
| FDK | 0.52*** | 0.34*** | 0.46*** | 0.53*** | 0.01 | -0.11 | - | IND |
| DON | 0.36*** | 0.07 | 0.25** | 0.20* | 0.20** | 0.23*** | - | FDK |
| FT | 0.12* | 0.18* | 0.21* | 0.00 | 0.09 | 0.41*** | - | DON |
| VASN 2009 | |  |  |  |  |  |  |  |
| SEV | 0.50*** | 0.60*** | 0.83*** | 0.38*** | 0.42*** | -0.39*** | -0.45*** | INC |
| IND | 0.76*** | 0.85*** | 0.84*** | 0.40*** | 0.25*** | -0.27** | -0.40*** | SEV |
| FDK | 0.46*** | 0.43*** | 0.53*** | 0.48*** | 0.37*** | -0.30*** | -0.51*** | IND |
| DON | 0.21* | 0.09 | 0.10 | 0.22** | 0.38*** | 0.16* | -0.71*** | FDK |
| FT | -0.18* | 0.00 | -0.06 | 0.17 | 0.23** | 0.09 | -0.25*** | DON |
| HT | -0.57*** | -0.55*** | -0.69*** | -0.49*** | -0.05 | 0.16* | -0.02 | FT |
| VAFLD 2009 | |  |  |  |  |  |  |  |
| SEV | 0.63*** | 0.33*** | 0.79*** | 0.19** | 0.28*** | 0.00 | -0.18** | INC |
| IND | 0.86*** | 0.82*** | 0.66*** | 0.01 | 0.08 | -0.14 | -0.03 | SEV |
| FDK | 0.28*** | 0.12 | 0.23** | 0.11 | 0.18** | -0.01 | -0.14 | IND |
| DON | 0.18* | 0.13 | 0.13 | 0.10 | 0.28*** | 0.36*** | -0.60*** | FDK |
| HD | 0.22** | 0.08 | 0.16 | 0.31*** | 0.34*** | 0.17* | -0.01 | DON |
| HT | -0.34*** | -0.35*** | -0.31*** | -0.32*** | 0.15 | 0.10 | -0.20* | HD |

^a^ B/M – F_7:14_ RIL from the cross of Becker/Massey, E/MO – F_11_ RILs from the cross of Ernie/MO 94-317

^b^ Abbreviations of traits: INC – Incidence (%), SEV – Severity (%), IND – Index (0-100), FDK – Fusarium damaged kernels (%), DON – Deoxynivalenol (mg kg^-1^), FT – Flowering time (d from Jan 1), HD – Heading date (d from Jan 1), HT – Height (cm)

^c, d^ FT was recorded at VASN in 2008 and 2009, HD was recorded at VAFLD in 2009, HT was not recorded at VASN in 2008

^e^ Abbreviations of environments: VA – Virginia, SN – Scab nursery, FLD – Field

^e^ *, **, *** mean significant at P < 0.05, 0.01, and 0.001

Supplementary Table S3 Marker alleles linked to quantitative trait loci and genes associated with FHB resistance in wheat parents of both Becker/Massey and Ernie/MO 94-317 populations

| Chromosome | Markers/genes | Massey | Ernie | Becker | MO94-317 | QTL^a^ | NUWWSN 2012 (60)^b^ | USFHBN 2012 (51)^c^ |
| --- | --- | --- | --- | --- | --- | --- | --- | --- |
| 2DS | *Ppd-D1* | 413^d^ | 413 | 284 | 284 | *Qfhs.vt-2DS*  *Qfhs.umc-2DS*  *Qfhs.vt-3BL, Qfhs.umc-3BL* | 43 | 22 |
|  | *Rht8c(Xgwm261)* | 164+175 | 164+175 | 192 | 166 |  | 5 | 0 |
| 3BL (GH SEV) | Xwmc418 | 263 | 263 | 271 | 270 |  | 15 | 15 |
|  | Xwmc827b  Xwmc471 | 214  262 | 214  261 | 195  219 | 209  223 |  | -^e^  7 | -  2 |
| 4BS | *Rht-B1* | *Rht-B1b* | *Rht-B1b* | *Rht-B1a* | *Rht-B1a* | *Qfhs.vt-4BS, Qfdk.vt-4BS, Qfhs.umc-4BS, Qfdk.umc-4BS* | 28 | 35 |
|  | Xgwm513 | 149 | 149 | 144 | 144 |  | - | - |
| 4DS | *Rht-D1* | *Rht-D1a* | *Rht-D1a* | *Rht-D1b* | *Rht-D1b* | *Qfhs.vt-4DS, Qfhs.umc-4DS, Qfdk.umc-4DS, Qdon.umc-4DS* | 42 | 23 |
|  | Xbarc334 | 166 | 173 | 164 | 170 |  | - | - |

^a^ Underlined QTL were associated with greenhouse severity, vt – population was developed at Virginia Tech, umc – population was developed at University of Missouri-Columbia

^b,c^ NUWWSN – Northern Uniform Winter Wheat Scab Nursery (60 entries), USFHBN – Uniform Southern Fusarium Head Blight Nursery (51 entries), both nurseries contain soft red winter wheat advanced lines from more than 10 wheat breeding programs in the Eastern U.S.

^d^ Marker allele size in bp was underlined for those favorable alleles in corresponding parental source

^e^ “-” means markers were not screened
